# Variant of X-Linked Chronic Granulomatous Disease Revealed by a Severe *Burkholderia cepacia* Invasive Infection in an Infant

**DOI:** 10.1155/2013/323614

**Published:** 2013-07-21

**Authors:** Saul Oswaldo Lugo Reyes, Nizar Mahlaoui, Carolina Prando, Lizbeth Blancas Galicia, Marjorie Hubeau, Stéphane Blanche, Capucine Picard, Jean-Laurent Casanova, Jacinta Bustamante

**Affiliations:** ^1^Immunodeficiencies Research Unit, National Institute of Pediatrics, Coyoacan, 04530 Mexico City, DF, Mexico; ^2^Laboratory of Human Genetics of Infectious Diseases, INSERM U980, University Paris Descartes, Paris Sorbonne Cité, 75014 Paris, France; ^3^French Reference Center for Primary Immune Deficiencies (CEREDIH), Necker-Enfants Malades University Hospital, AP-HP, 75015 Paris, France; ^4^Pediatric Immunology-Hematology Unit, Necker-Enfants Malades University Hospital, AP-HP, 75015 Paris, France; ^5^St. Giles Laboratory of Human Genetics of Infectious Diseases, Rockefeller University, New York, NY 10065, USA; ^6^Bioinformatics Laboratory, Pelé Pequeno Principe Research Institute, 80250-060 Curitiba, PR, Brazil

## Abstract

Chronic granulomatous disease (CGD) is a primary immunodeficiency characterized by increased susceptibility to bacteria and fungi since early in life, caused by mutations in any of the five genes coding for protein subunits in NADPH oxidase. X-linked variant CGD can be missed during routine evaluation or present later in life due to hypomorphic mutations and a residual superoxide production. The case of a 10-month-old boy who died of pneumonia is reported. The isolation of *Burkholderia cepacia* from his lung, together with a marginally low nitroblue tetrazolium reduction assay (NBT), made us suspect and pursue the molecular diagnosis of CGD. A postmortem genetic analysis finally demonstrated CGD caused by a hypomorphic missense mutation with normal gp91^*phox*^ expression. In a patient being investigated for unusually severe or recurrent infection, a high index of suspicion of immunodeficiency must be maintained.

## 1. Introduction

Chronic granulomatous disease (CGD) is a rare primary immunodeficiency that affects microbial killing by phagocytes, resulting in bacterial, fungal, and/or mycobacterial infections since early life [1, 2]. The superoxide production by NADPH oxidase is markedly reduced or absent due to mutations in any of the five genes coding for protein subunits of the enzymatic complex [3]. Mutations in *CYBB*, coding for gp91^*phox*^, result in the most common X-linked CGD (65%–70% of all cases) [4]. Hypomorphic mutations (Xgp91^+^ and Xgp91^−^) may result in X-linked variant CGD [5, 6]. Patients with variant CGD express the gp91^*phox*^ protein and produce decreased but detectable superoxide, which allow the defect to manifest later in life with a milder history of infections. By far, the most common micro-organisms causing infections in CGD are *Staphylococcus aureus* and *Aspergillus* species; other agents include *Pseudomonas*, *Serratia*, *Salmonella*, and *Candida* species. *Burkholderia cepacia* infection is frequently associated to CGD diagnosis (6–8). Here, we present the case of a patient who died of *Burkholderia cepacia* lung infection, in whom the diagnosis of X-CGD could only be attained postmortem due to residual superoxide production and normal protein expression.

## 2. Case Report

A 10-month-old boy, the first child of nonconsanguineous parents living in the Tahiti archipelago (French Polynesia), was referred for severe pneumonia. The father is from Europe and the mother is from Oceania; there was no relevant family history. During the first months of life, the patient had experienced some infections, mostly of the upper airways, as well as bronchitis and diarrhea. He received all the immunizations according to his age (including BCG) with no adverse events. He developed a failure to thrive at the age of 3 months. One month before admission he had a severe lung infection with fever, cough, dyspnea, and diarrhea, unresponsive to an empiric oral macrolide (josamycin). Upon admission to his local hospital, he had fever (39.5°C), mild respiratory distress, and crackles on auscultation. Oxygen saturation was 95% in room air. Complete blood count (CBC) reported marked leukocytosis (36,600/mL) with neutrophilia (29,000 polymorphonuclear cells (PMN)/mL) and anemia (Hb = 7.6 g/dL); serum immunoglobulin levels were as follows: IgG = 1,900 mg/dL (reference value for 7–12 months: 661 ± 219 mg/dL), IgA = 166 mg/dL (37 ± 18), IgM = 220 mg/dL (54 ± 23), and IgE 43 IU/mL (normal < 20 IU/mL). Chloride sweat test and tuberculin skin test were negative. Chest X-ray and computed tomography scan (CT) revealed bilateral pneumonia with multiple excavations in both lungs. Intravenous (IV) cefotaxime and fosfomycin were started for suspected staphylococcal pneumonia. Bronchoscopy showed diffuse edema of the trachea and bronchi. Bronchoalveolar lavage (BAL) and Gram stain reported 1,100 cells (97% PMN) and abundant Gram negative bacteria that grew *Burkholderia cepacia* (10^7^ CFU, >25 white cells). Antibiotherapy was then switched to IV rifamycin and trimethoprim/sulfamethoxazole. 

After a transient improvement, the patient's condition deteriorated, and he was referred to our hospital, where he was found to be small for his age and cachectic, with severe respiratory distress and hepatosplenomegaly. Lung CT scan revealed extensive destruction of the lungs with multiple bullous lesions and opacification of the left lung; the right lung had multiple nodular lesions and opacified upper lobe. Immunological workup confirmed marked leukocytosis with neutrophilia and anemia, elevated serum C-reactive protein (CRP = 165 mg/L), and fibrinogen (6 g/L). BAL retrieved *Burkholderia cepacia* (10^6^ CFU/mL, >25 white cells/field). Lymphocyte subset counts, T lymphocytes proliferation, and specific antibody production assays were all normal. Nitroblue tetrazolium reduction (NBT) test and luminol chemiluminescence to assess reactive oxygen species (ROS) production in PMNs repeatedly showed a baseline activity level at around 45% (low but detectable), and response to stimulation was poor. Chemotaxis chamber assay was normal, as well as CD18 and CD11a,b,c expression on PMNs. When a peripheral blood smear reported vacuolized enlarged PMNs, dense granule disease was suspected and ruled out: normal secretory vesicles, secondary granules, azurophile granules, and myeloperoxidase production; normal specific staining of secondary granule proteases (neutrophil elastase, myeloperoxidase, Cathepsin G, and Lactoferrin). Despite intensive supportive care, including broad-spectrum antibiotics and daily granulocyte transfusions, his lung infection worsened, and he finally died of acute respiratory distress and multiorgan dysfunction in the intensive care unit. Permission to perform an autopsy was refused by his parents.

**Figure 1:**
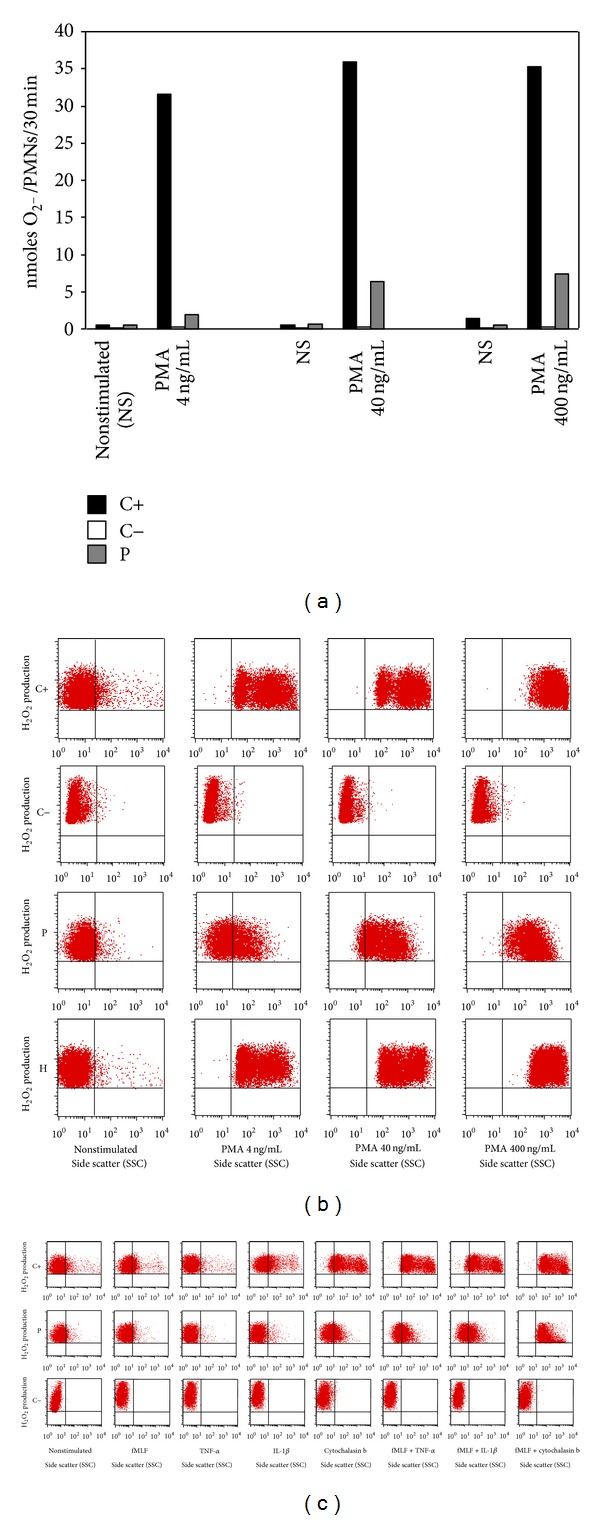
NADPH oxidase activity evaluation in PMNs. (a) Superoxide generation was measured by assaying superoxide dismutase-inhibitable cytochrome-*c* reduction in PNMs after adding three doses of PMA (4, 40, and 400 ng/mL), for healthy controls (C+), CGD patient (C−), and our patient (P). (b) Histograms for the flow cytometric analysis of intracellular H_2_O_2_ production, using the fluorescent 123DHR probe in PMNs from a healthy control (C+), an X-linked CGD patient (C−), the proband (P), and the mother (H), before (NS) and after stimulation with PMA (4, 40, and 400 ng/mL). (c) PMNs from C+, C−, and P were left untreated or treated with TNF-*α*, IL-1*β*, and cytochalasin b and then stimulated with fMLF. The results shown are representative of two independent experiments.

The clinical presentation and the impaired NBT reduction assays of this boy were consistent with a primary phagocyte defect. We assessed superoxide (O_2_^−^) production in PMNs from the patient as measured by the cytochrome-*c* reduction assay, compared to another patient with known X-linked CGD (−) and a healthy control (+), following stimulation with phorbol myristate acetate (PMA). Residual NADPH oxidase activity was detected in the PMNs of the patient (Figure 1(a)). In addition, 123-dihydrorhodamine (DHR) oxidation assay by flow cytometry revealed a partial deficiency of ROS production in the patient's PMN, while his mother had two granulocyte populations: one strongly rhodamine-positive (reactive) and the other rhodamine-low fluorescence intensity (Figure 1(b)). These results again suggested that our patient had a partial defect in the respiratory burst. We next investigated the H_2_O_2_ production upon milder activation, involving priming with TNF-*α*, IL-1*β*, or cytochalasin b, followed by fMLF (formyl-methionyl-leucyl-phenylalanine) stimulation. PMNs from the patient produced detectable but low H_2_O_2_ (Figure 1(c)).

**Figure 2:**
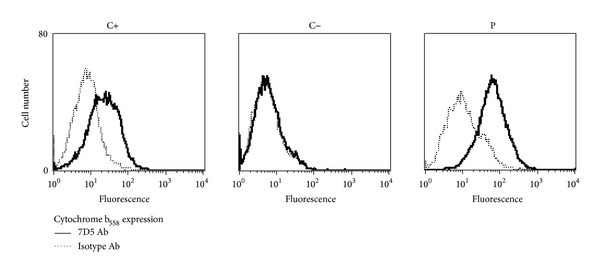
Expression of gp91^*phox*^ in a patient with the H338R *CYBB* mutation. Immunostaining of cytochrome b_558_ in EBV-B cells from a healthy control (C+), an X-linked CGD patient (C−), and the patient (P). Cell surface staining with mAb7D5 (an antibody specific for the extracellular epitope of gp91^*phox*^; solid lines); an isotype IgG1 (dotted lines) followed by staining with an Alexa Fluor 488 goat anti-mouse Ig secondary antibody. The results shown are representative of two independent experiments.

Genomic sequencing of *CYBB* revealed a hemizygous A > G substitution in exon 9, generating the replacement of a histidine by an arginine residue (H338R) in the FAD binding domain (FADBR), a probably damaging substitution according to the PolyPhen-2 prediction website (http://genetics.bwh.harvard.edu/pph2/). The patient's mother was heterozygous, and his brother (born after the patient's death) was hemizygous for the mutation. The mutation was confirmed also in cDNA from the patient (c.1013A > G). We investigated the molecular basis of the germline H338R mutation through detection of flavocytochrome b_558_ expression by flow cytometry, using the monoclonal antibody 7D5 (MBL, Nagasaki, Japan), which recognizes residues ^160^IKNP^163^ and ^226^RIVRG^230^ on gp91^*phox*^ in the presence of p22^*phox*^. Protein expression in Epstein-Barr virus transformed B cells (EBV-B cells) from the patient was similar to the healthy control (Figure 2).

## 3. Discussion

The isolation of *Burkholderia cepacia* from lung secretion or blood of a previously healthy patient is strongly suggestive of CGD. Aside from it, lung infections caused by *Burkholderia *species can be seen in patients with existing bronchiectasis (lung epithelial damage is a prerequisite for *Burkholderia *invasiveness), including notably patients with cystic fibrosis [7] and in some immunocompromised and hospitalised patients [8, 9]. In a child being investigated for recurrent infections, isolation of *Burkholderia *should always raise the suspicion of CGD [10–12]. For some patients with normal gp91^*phox*^ expression and residual superoxide production as measured by conventional assays, a milder activation assay with fMLF might be needed to demonstrate low ROS production. 

Missense mutations beyond aminoacid 309 of gp91^*phox*^ usually allow normal protein expression but result in null superoxide production. The patient's residual ROS generation is thus different from the thorough survival analysis by Kuhns et al. [3]. Also, given this infant's residual superoxide production, a severe course with early demise is surprising.

In conclusion, we identified *postmortem *a point mutation in a CGD causing gene from a 10-month-old boy who presented with a *Burkholderia* spp. overwhelming lung infection. X-CGD diagnosis was delayed because of initial normal results. A high index of suspicion for CGD must be maintained in patients with *Burkholderia* isolates and close to normal values of usual CGD diagnostic tests such as NBT. An early and accurate diagnosis can lead to genetic counselling, to family screening, and to a timely intervention.

## Abbreviations

CGD: Chronic granulomatous disease
PID: Primary immune deficiency
NADPH: Nicotinamide adenine dinucleotide phosphate hydrogen
BCG: Bacillus Calmette-Guérin.
